# SPO11-C631T Gene Polymorphism: Association With Male Infertility and an in Silico-Analysis 

**Published:** 2015-11

**Authors:** Mohammad Karimian, Hossein Nikzad, Abolfazl Azami-Tameh, Aliakbar Taherian, Fatemeh Zahra Darvishi, Mohammad Javad Haghighatnia

**Affiliations:** 1 Gametogenesis Research Center, Kashan University of Medical Sciences, Kashan, Iran; 2 Anatomical Sciences Research Center, Kashan University of Medical Sciences, Kashan, Iran; 3 Department of Biology, Division of Biochemistry, Cell and Molecular Biology, University of Isfahan, Isfahan, Iran

**Keywords:** Male Infertility, SPO11 Gene, Single Nucleotide Polymorphism, PCR-RFLP

## Abstract

**Objective:** To investigate the association of C631T single nucleotide polymorphisms in *SPO11* gene with male infertilityfollowed by an *in silico* approach. *SPO11* is a gene involved in meiosis and spermatogenesis process, which in humans, this gene is located on chromosome 20 (20q13.2-13.3) with 13 exons.

**Materials and methods:** In a case-control study, 200 blood samples were collected from the IVF center (Kashan, Iran) including; 100 infertile and 100 healthy control men. *SPO11*-C631T were genotyped using polymerase chain reaction-restriction fragment length polymorphism (PCR-RFLP) method.The effects of C631T transition on the structure of mRNA and protein of *SPO11* was evaluated by bioinformatics tools.

**Results:** Our data revealed that all subjects were wild-type homozygous inC631T positionsand just a sample from fertile group was heterozygousin C631T (OR: 0.3300, 95% CI: 0.0133 to 8.1992, p = 0.4988).Our *in silico*-analysis revealed that C631T transition could make fundamental changes in the structure of the mRNA (Score: 0.1983) and protein (PROVEAN Score: -3.371; Reliability Index: 4; Expected Accuracy: 82%) of *SPO11*. Also, C631T substitution could change the aggregation prone regions of the *SPO11* protein (dTANGO = 209.99).

**Conclusion:** So even though the* SPO11*-C631T don’t increase the risk of male infertility, it could be deleterious for themRNA and protein.

## Introduction

Infertility affects 15% of couples. Approximately half of Infertility causes are due to male factors (1, 2) and about 15%of these cases are genetic abnormalities including, chromosomal aberrations (3, 4), chromosomal translocation (5), Y chromosome microdeletions (6), mutations in single-genes such as CFTR, INSL3, and androgen receptor genes (7, 8). Deleterious genepolymorphisms in key genes involved in spermatogenesis such as folate metabolizing genes,telomere processing genes, and protamine genes may be responsible forthe reduced sperm count and poor sperm quality (9-11).

**Figure 1:**
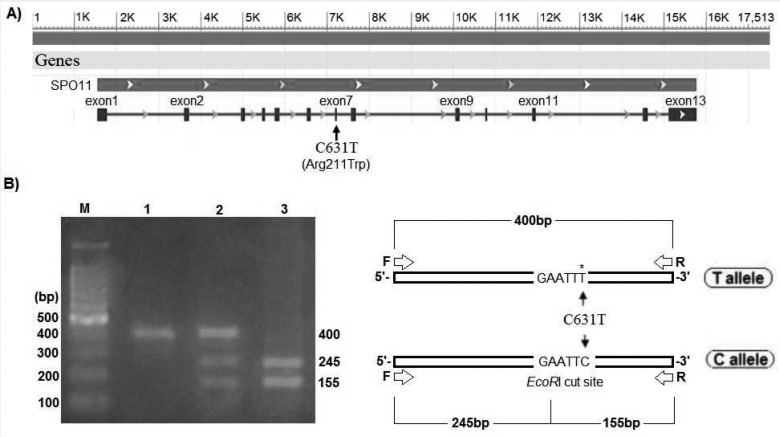
Location and genotyping of C631T. A) Human SPO11 gene map was deduced from NCBI data bank, which contain 13 exon, the location of C631T SNP on exon 7, was shown by arrowhead; B) Schematic and results of the PCR-RFLP. Digestion map for 400-bp PCR fragment by EcoRI enzyme [lane M = DNA marker; lane1 = PCR product as a control; lane2 = the sample with CT genotype containing 400-bp, 245-bp and 155-bp bands; lane3 = the sample with CC genotype containing 245-bp and 255-bp bands].

A possible candidate for genetic susceptibility to spermatogenic failure is the *SPO11*gene. The human *SPO11* gene consists of 13 exons and is located on 20q13.2-13.3 (Figure 1A) (12). This gene encodes a critical protein with 396 amino acids. Two major *SPO11* isoforms are generated by alternative splicing: *SPO11*α (exon 2 skipped) and *SPO11*β.*SPO11* is a critical protein in meiosis process. Recombination during meiosis increases genetic diversity, therefore the central purpose of recombination is to promote accurate segregation of homologous chromosomes to generate haploidcells (13). During meiosis, programmed double-strand breaks (DSBs) occur, which leads to interhomolog recombination. Meiotic DSB repair is remarkablyrobust, which exist in every cell to repair spontaneous DNA damage.Meiotic recombination mechanisms have been deduced primarily from studies in budding yeast (14-16). DSBs are performed by the *SPO11*molecule, and then the molecule is removed from DNA, the 5'ends are cleaved to generate 3' single-stranded tails (17).* SPO11*acts as adimer, and one molecule remains covalently attached to each DSB end up on generation of the break (18). The catalytic activity for DSBs formation seemsthat *SPO11* presumably acts as a transesterase rather than as an endonuclease (19). There is a tyrosine residue at active site of the protein that has a crucial role in DSB(18). Yeast *SPO11* mutants show a range of phenotypes frompartial loss of function, to complete loss of DSB formation. Varying levels of synaptonemal complex (SC) defects are also observed, highlighting the importance of DSB formation inhomologous chromosome synapsis. A previousstudy investigated the association of five single nucleotide polymorphisms (SNPs) in *SPO11* gene with male infertility (20). These five SNPs included rs28368062, rs28368064, rs79564060, rs23736832 and rs28368082. The rs28368082 (C631T) SNP leads to Arginine to Tryptophan substitution at codon 211 (Arg211Trp) located on exon7 (Figure 1A). In the present study, we investigated the association of C631T substitution in *SPO11* gene with male infertility followed by a novel *in silico*-analysis.

## Materials and methods

In a case–control study, all subjects were randomly selected from the IVF center (Kashan, Iran). The infertile patients were selected from couples attending the infertility clinic who had a history of infertility above 1 year. After signing an informed consent, a detailed medical and reproductive history was obtained from all subjects, including reproductive history and infertility evaluation of the female partner. Samples were first screened for genetic and familial diseases.100 patients were finally selected as idiopathic infertile case group. 100healthy fertile male volunteers served as the control group. The semen analysis for sperm concentration, morphology, and motility performed following the World Health Organization (WHO) criteria (21). Finally 2 ml blood was taken from each individual and preserved at -20°C. All the participants’ informed written consent and the study was approved by the Medical Research Ethics Committee of the Kashan University of Medical Sciences on 30 August 2011 (reference number 9254).

***SNP genotyping***

The polymerase chain reaction-restriction fragment length polymorphism (PCR-RFLP) method used to *SPO11*-C631T genotyping. To design specific primers for PCR-RFLP, the genomic sequences of human *SPO11* obtained from National Center for Biotechnology Information (NCBI, http://www.ncbi.nlm.nih.gov/nucleotide). Specific primers designed by GeneRunner software (Ver. 3.05 / 4.0.9.3 Beta). The forward primer sequence was: 5'-AGTACTAAACTTAGTACCCCTG-3' and the reverse primer sequence was: 5'-ACTAAAGAAGGGACCATGGTGT-3'. The primers location and C631T site has been shown in figure1B. Primers were ordered from CinnaGen Company (Iran). The region containing C631T was amplified by PCR method in thermal cycler system (peqSTAR, Germany). To amplify the *SPO11*μ*Taq*μμ_2, _0.35 µM each of forward and reverse primers, and 20 ng of template DNA (All PCR reagents were purchased from Fermentas, Germany). PCR performed with the following program: The initial denaturation at 94˚C for 10 minutes followed by 33 repetitive cycles including, denaturation temperature: 94˚C for 30seconds; annealing temperature: 57˚C for 30 seconds, extension temperature: 72˚C for 30seconds and final extension temperature: 72˚C for 5 minutes. Then The PCR products digested with *EcoR*I (Fermentas Co., Leon-Rot, Germany) at 37˚C for 16h and analyzed by 1% agarose gel electrophoresis and staining with GelRed™ (Biotium, Hayward, CA, USA).

***In silico-analysis***

DNA sequences of *SPO11* gene obtained from NCBI (Accession No. AY957583.1). The coding sequence domain of *SPO11* translated by ExPASy server (http://web.expasy.org/translate/). The physicochemical properties of the protein was analyzed using ProtParam server (http://web.expasy. org/protparam/). The secondary structure of the *SPO11* assessed by bioinf server (http://bioinf.cs.ucl.ac.uk/psipred/) (22). The three-dimensional structure of the protein was assessed using Itasser (http://zhanglab.ccmb.med.umich.edu/I-Tas.SER) (23). Accelrys DS Visualiserver.4.0 (accelrys company, http://accelrys.com/products/ discovery-studio/visualization.php) applied to investigate the three-dimensional structure, hydrophobicity, and Ramachandran plot of *SPO11*protein. The effect of Arg211Trp substitution on protein functions evaluated by SNAP (Screening for Non-Acceptable Polymorphisms; https://rostlab.org/services/snap/) (24), PolyPhen-2 (http://genetics.bwh.harvard.edu/pph2/) (25), and SIFT (Scale-invariant feature transform; http://sift.jcvi.org/) (26) servers. The effect of C631T substitution on the mRNA structure assessed by RNAsnp Web Server (http://rth.dk/resources/rnasnp/) (27). SNPeffect 4.0 server (http://snpeffect. switchlab.org/) (28) was used for detection of Arg211Trp effects on the chaperone binding tendency, amyloid-forming regions, and aggregation prone regions in the protein sequence.For the CT genotype, odd ratios (ORs) and 95% confidence interval (95% CI) were calculated. Differences between infertile and healthy groups were assessed by Chi-square Test. Statistical analyses performed by the SPSS ver. 16 (SPSS, Chicago, IL).

## Results

***Analysis of C631TSNP of SPO11***

Human genome was extracted and showed a major band with low mobility on agarose gel. However, using the extracted genome as template, *SPO11* gene containing C631T single nucleotide polymorphism with the approximate size 400 bp was amplified by utilizing the conditions which were described in materials and methods section. *EcoR*I cuts only the C allele, which resulted in 245 bp and 155 bp fragments. All PCR products of *SPO11* fragment (400 bp) containing C631T were digested by *EcoR*I. The absence of polymorphism in C631T causes full enzymatic digestion of amplified fragment whereas heterozygote samples produced 3 bands on agarose gel (Figure1B). In 199 samples in the present study, *EcoR*I produced two fragments with aforementioned lengths. Only one sample from control group was heterozygote in C631T (OR: 0.3300, 95% CI: 0.0133 to 8.1992, p = 0.4988).

***Structural analysis***

The physicochemical properties of *SPO11* protein, determined using ProtParam, indicated that the *SPO11* protein contains 396 amino acids. The normal known *SPO11* protein has a predicted molecular formula of C_1988_H_3195_N_539_O_576_S_21_, molecular mass of 44536.7 Da and an isoelectric point (pI) of 9.05. In the normal protein, the total number of negatively charged residues (Asp and Glu) is 41 and the total number of positively charged residues (Arg and Lys) is 50. The aliphatic index of the *SPO11*protein had been calculated to be 96.77. The instability index of the *SPO11* protein in normal was determined to be 59.99. The Grand average of hydropathicity (GRAVY) was calculated -0.088 for normal *SPO11*. The estimated half-life of the *SPO11* proteinin normal is 30h (mammalian reticulocytes, *in vitro*), (Expasy/ProtParam server). However these parameters for 631T phenotype summarized in table 1.

**Figure 2:**
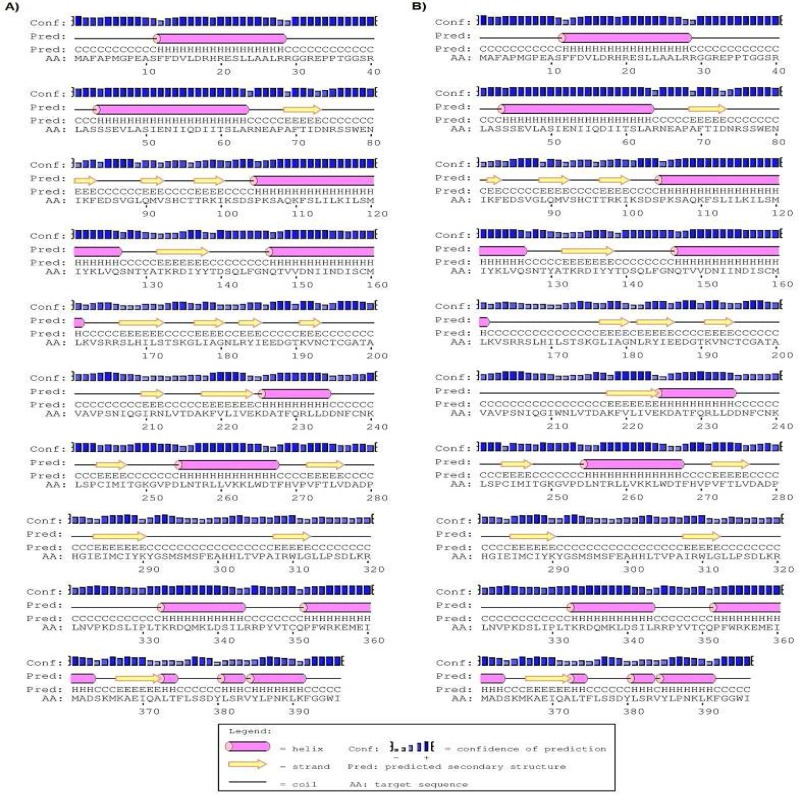
Secondary structure of the SPO11 protein, as determined using the Bioinf server. A) 631C phenotype; B) 631T phenotype. The changes between two phenotypes are evident in lines 5 and 6.

**Figure 3:**
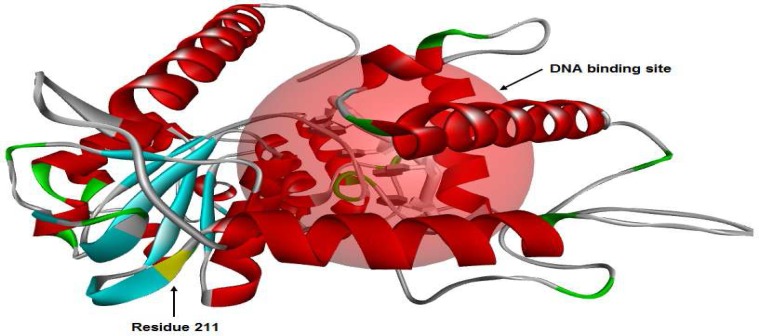
DNA binding site of SPO11 protein. Residue 211 and ligand were shown by arrowheads. The residue 211 located in a beta-sheet motif on the surface of the protein.

**Figure 4:**
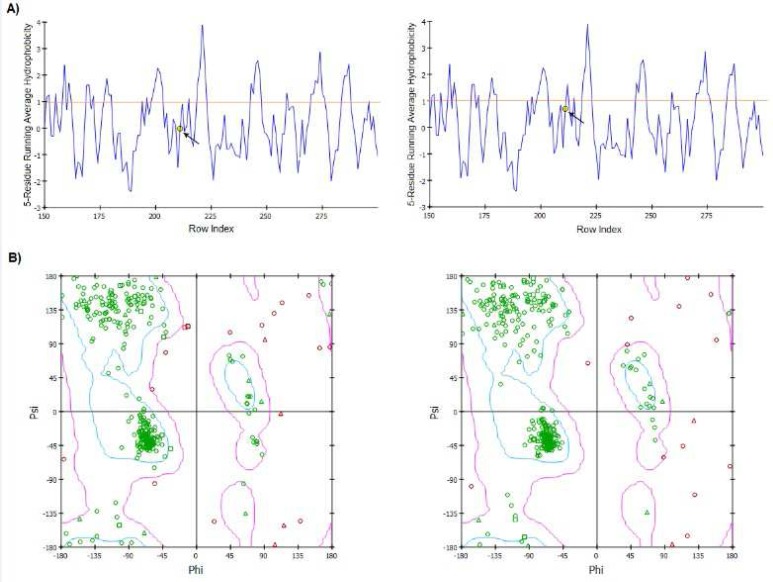
Hydrophobicity and Ramachandran plots for 631C and 631T phenotypes. A) Average hydrophobicity is -0.02 for Arg211 (right) and 0.7 for Trp211 (left). The residue211 shown by yellow dot; B) Ramachandran plot for 631C phenotype (left) and 631T phenotype (right).

**Figure 5:**
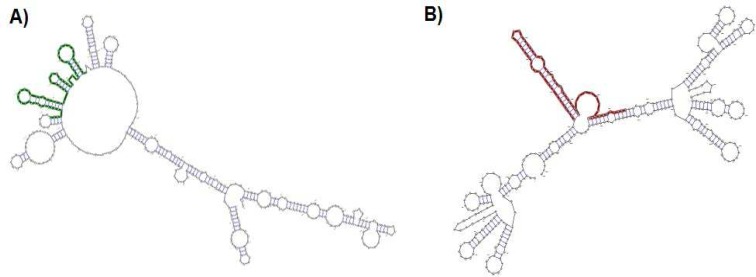
Secondary structure of SPO11. The secondary structure of wild-type mRNA with minimum free energy = -85.40 kcal/mol (right) and Mutant mRNA with minimum free energy = -86.50 kcal/mol (left).

The secondary structure of the *SPO11* protein evaluated by the Bioinf server (Figure 2). Our data revealed that the secondary structure of the *SPO11* protein differs between 631C and 631T phenotypes, especially in the helix, strand and coil motifs.Analysis of three-dimensional structure of *SPO11* protein by Accelrys DS Visualiser 4.0 revealed that residue 211located in a beta-sheet motif at the surface of *SPO11* (Figure 3). Additionally, the data obtained by a Ramachandran plot confirmed the structural stability for both 631C and 631T phenotypes of the protein (Figure 4A) Also data revealed that the peak of the hydrophobicity for 631Cwas -0.02 whereas this index shift to 0.7 for 631T (29) (Figure 4B). Analysis of C631T transition bySNPeffect server indicated that C631T does not affect the chaperone binding tendency (dLIMBO: 0.00) and amyloid-forming regions (dWALTZ: -27.61) whereas the substitution increases aggregation prone regions in *SPO11* protein (dTANGO: 1107.96).Data from SNAP server indicated that the C631T substitution may have damaging effects in *SPO11* structure (Prediction: Non-neutral, Reliability Index: 3, Expected Accuracy: 78%) consistent the data from SIFT server (PROVEAN score: -3.371, Prediction (cutoff = -2.5): Deleterious). While the data from PolyPhen2 showed that C631Thave a low probability of damaging (Score: 0.014, Sensitivity: 0.96, Specificity: 0.79). Predicting C631T effects on local RNA secondary structure of *SPO11* revealed that the SNP make fundamental changes on the secondary structure of mRNA (Distance: 0.1080, P-value: 0.1983; the P-value less than 0.2 is significant structural change) (Figure 5).

## Discussion

*SPO11* is a critical gene and is involved in the recombination process of genes in meiosis (30, 31). Although many studies have been done on the biological role of *SPO11* gene but its role in infertility has not been studied extensively yet. A common single nucleotide polymorphismin *SPO11* changes a cytosine residue to thymine (631C→T) which leads to Arginine to Tryptophan substitution at codon 211 in *SPO11* protein. In this study, firstly we investigate the association of 631C→T with male infertility in an Iranian population. Our results showed that only one of the fertile subjects had CT genotype in C631T location and the rest of the subjects (100 infertile and 99 fertile men) had CC genotype. 

**Table 1:** Physicochemical properties for wild type, 106G and 631T phenotype of SPO11 protein

Protein Phenotype	Molecular weight	Theoretical pI	Estimated half-life	Instability index	Aliphatic index	GRAVY^a^
Wild	44536.7	9.05	30 hours	59.99	96.77	-0.088
631T	44566.7	8.97	30 hours	59.99	96.77	-0.079

a Grand average of hydropathicity

In 2005 some SNPs was studied in 48 male azoospermia and 48 oligospermia in USA. Results showed that one of the azoospermi subject had a glutamate changed to lysine (Glu9Lys) in exon one another had an aspartate changed to glutamate (Asp277Glu) (32). In 2011 the relation of five different SNPs (rs28368062, rs28368064, rs79564060, rs2373683 and rs28368082) with infertility was studied in chines men.Only one of the SNPs (rs28368082) was related to infertility and the incidence of CT genotype in rs28368082 location in infertile men was higher than fertile men (p = 0.023). Also the frequency of T allele in rs28368082 location was higher in infertile than fertile men as well (p = 0.025) (20). In 2014 Ghalkhani et al. performed another study in Iranian population. They recruited a total of 113 infertile men, including: 58 azoospermic men and 55 oligozoospermic men. They selected the subjects from three regions; including: North (19 azoospermia and 17 oligozoospermia), Center (20 azoospermia and 20 oligozoospermia), and South (19 azoospermia and 18 oligozoospermia) of Iran. They reported that C361T was significantly associated with male infertility in all populations except oligozoospermic cases from the Center region (33). The inconsistency between the studies could arise from to race, geography or genetic differences of the study population.

In meiosis, recombination of genes occurs when homolog chromosomes separate from each other to produce haploid gametes (34, 35). The expression of *SPO11* gene increases during meiosis (36). In yeast, by breaking the double strand DNA, *SPO11* facilitate the process of recombination. It been shown that in rats, a disrupted *SPO11* gene can causes male infertility (37). Any disturbance in the recombination process of meiosis counteracts the proper dissociation of the homolog chromosomes and leads to aneuploidy and making defected gametes. Therefore any change or mutation in *SPO11* gene can cause infertility in men (17, 38).

Effects of missense mutations on protein features using*in silico*prediction can be determined even without a well-defined protein structure (39). Study the secondary structure of *SPO11* showed that Arg211Trp substitution makes some changes in the structure of the coils, strands, and helixes in the protein (Figure 2).The physicochemical properties of *SPO11* protein, detected byProtParam server, showed that the *SPO11* protein contains 396residues. The wildphenotype of*SPO11* has a predicted molecular formula of C_1988_H_3195_N_539_O_576_S_21_, molecular mass of 44536.7 Da and an isoelectric point (pI) of 9.05; the molecular mass of the 631T phenotype increases to 44566.7 Da. Because we found that Arg had five carbon atom less than Trp, the molecular weight of the normal* SPO11* protein is lower than that of themutant protein resulting in an increase in the ratio of pI to MW for mutant protein. In the normal protein, the total number of negatively charged residues (Asp and Glu) is 41 and the total number of positively charged residues (Arg and Lys) is 50 whereas these ratios in mutant protein are 41 and 49 respectively. Therefore pI shifts from 9.05 for normal protein to 8.97 for mutant *SPO11*. The estimated half-life of the *SPO11* proteinin normal is 30 h (mammalian reticulocytes, *in vitro*). The hydrophobic pattern, determined using the Kyte and Doolittle hydrophobicity scale (29), showed that the average hydrophobicity of Arg211 in the normal protein equals -0.02 whereas this index shifts to 0.7for Trp211 in the mutant protein (Figure 4A). Therefor the Trp211 as a hydrophobic residue tends to cluster in the interior of the molecule that this change may be deleterious for the protein (40). SIFT operates by utilizing both physical properties of amino acids and sequence homology of variedorthologus. The function of PolyPhenis based on physical and comparative approaches to evaluate the impact of an amino acid substitution on the function and structure of a protein. SNAP prediction is based on evolutionary information, biophysical properties of substitution and structure of protein and annotations (39, 41). The data from SNAP and SIFT showed that C631T might be deleterious for function and structure of *SPO11*. Whereas PolyPhen predicted the C631T transition as a benign substitution. Analysis of C631T substitution by SNPeffect server indicated that the substitution increases aggregation prone regions in *SPO11 *protein (dTANGO: 1107.96). The total TANGO score for the protein is 1107.96. Mutations can increase (dTANGO > 50), decrease (dTANGO < -50) or not affect aggregation propensity (dTANGO between -50 and 50). For C6311 substitution, dTANGO equals 209.99 which means that the mutation increases the aggregation tendency of *SPO11* protein. mRNAs containing different nucleotide at SNP position may vary in their interactions with cellular molecules involved in mRNA transport, maturation, translation, or degradation (42). Analysis the effects of C631T transition on secondary structure of mRNA showed that the SNP could alter the structure and minimum free energy of mRNA. Therefore C631T might alter the *SPO11* gene expression.

Due to the effects of gene–gene interactions and environmental factors, we need a larger group of case and control in order to obtain accurate data. Although our *in silico-*analysis lead to better understanding of the effects of C631T on the *SPO11*, but more studies are essential to evaluate the influence of the environmental factor on sperm parameters of men with different *SPO11* variants.

## Conclusion

According to our research, although C631T polymorphism in the *SPO11* gene is not associated with idiopathic male infertility in the Iran; Kashan population, but *in silico*-analysis of *SPO11*indicated that the SNP could make fundamental changes in the protein and mRNA structure of *SPO11*. Therefore further studies with larger sample size and diverse ethnic populations are required to confirm the general validity of our findings. Since we only studies one SNP, further studies are required to investigate the possible effects of other mutations (alone or in combination) on *SPO11* function. Also functional studies on these polymorphisms are needed to clarify the molecular mechanisms of the observed results.

## References

[1] Huynh T, Mollard R, Trounson A (2002). Selected genetic factors associated with male infertility. Hum Reprod Update.

[2] Campagne DM (2013). Can Male Fertility Be Improved Prior to Assisted Reproduction through The Control of Uncommonly Considered Factors. Int J Fertil Steril.

[3] Dohle GR, Halley DJ, Van Hemel JO, van den Ouwel AM, Pieters MH, Weber RF (2002). Genetic risk factors in infertile men with severe oligozoospermia and azoospermia. Human Reproduction.

[4] Jaffe SB, Jewelewicz R (1991). The basic infertility investigation. Fertility and Sterility.

[5] Chandley AC, Edmond P, Christie S, Gowans L, Fletcher J, Frackiewicz A (1975). Cytogenetics and infertility in man. I. Karyotype and seminal analysis: results of a five-year survey of men attending a subfertility clinic. Annals of Human Genetics.

[6] Suganthi R, Vijesh VV, Vandana N, Fathima Ali Benazir J (2014). Y choromosomalmicrodeletion screening in the workup of male infertility and its current status in India. International Journal of Fertility & Sterility.

[7] Mosaad YM, Shahin D, Elkholy AA, Mosbah A, Badawy W (2012). CAG repeat length in androgen receptor gene and male infertility in Egyptian patients. Andrologia.

[8] Schulz S, Jakubiczka S, Kropf S, Nickel I, Muschke P, Kleinstein J (2006). Increased frequency of cystic fibrosis transmembrane conductance regulator gene mutations in infertile males. Fertility and Sterility.

[9] Mfady DS, Sadiq MF, Khabour OF, Fararjeh AS, Abu-Awad A, Khader Y (2014). Associations of variants in MTHFR and MTRR genes with male infertility in the Jordanian population. Gene.

[10] Yan L, Wu S, Zhang S, Ji G, Gu A (2014). Genetic variants in telomerase reverse transcriptase (TERT) and telomerase-associated protein 1 (TEP1) and the risk of male infertility. Gene.

[11] Salamian A, Ghaedi K, Razavi S, Tavalaee M, Tanhaei S, Tavalaee M (2008). Single nucleotide polymorphism analysis of protamine genes in infertile men. International Journal of Fertility & Sterility.

[12] Romanienko PJ, Camerini-Otero RD (1999). Cloning, characterization, and localization of mouse and human SPO11. Genomics.

[13] Handel MA, Schimenti JC (2010). Genetics of mammalian meiosis: regulation, dynamics and impact on fertility. Nature Reviews Genetics.

[14] Baudat F, de Massy B (2007). Regulating double-stranded DNA break repair towards crossover or non-crossover during mammalian meiosis. Chromosome Res.

[15] Bishop DK, Zickler D ( 2004). Early decision: meiotic crossover interference prior to stable strand exchange and synapsis. Cell.

[16] Keeney S, Neale MJ (2006). Initiation of meiotic recombination by formation of DNA double-strand breaks: mechanism and regulation. Biochemical Society Transactions.

[17] Cole F, Keeney S, Jasin M (2010). Evolutionary conservation of meiotic DSB proteins: more than just SPO11. Genes & Development.

[18] Inagaki A, Schoenmakers S, Baarends WM (2010). DNA double strand break repair, chromosome synapsis and transcriptional silencing in meiosis. Epigenetics.

[19] Barchi M, Jasin M (2003). Seeking new meiotic genes. Proc Natl Acad Sci USA.

[20] Zhang J, Zhou DX, Wang HX, Tian Z (2011). An association study of SPO11 gene single nucleotide polymorphisms with idiopathic male infertility in Chinese Han population. Journal of Assisted Reproduction and Genetics.

[21] (1999). World Health Organization Laboratory Manual for the Examination of Human Semen and Sperm–Cervical Mucus Interaction.

[22] Buchan DW, Minneci F, Nugent TC, Bryson K, Jones DT (2013). Scalable web services for the PSIPRED Protein Analysis Workbench. Nucleic Acids Research.

[23] Roy A, Kucukura A, Zhang Y (2010). I-TASSER: a unified platform for automated protein structure and function prediction. Nature Protocols.

[24] Bromberg Y, Rost B (2007). SNAP: predict effect of non-synonymous polymorphisms on function. Nucleic Acids Research.

[25] Adzhubei IA, Schmidt S, Peshkin L, Ramensky VE, Gerasimova, A, Bork P (2010). A method and server for predicting damaging missense mutations. Nature Methods.

[26] Kumar P, Henikoff S, Ng PC (2009). Predicting the effects of coding non-synonymous variants on protein function using the SIFT algorithm. Nature Protocols.

[27] Sabarinathan R, Tafer H, Seemann SE, Hofacker IL, Stadler PF, Gorodkin J (2013). The RNAsnp web server: predicting SNP effects on local RNA secondary structure. Nucleic Acids Research.

[28] De Baets G, Van Durme J, Reumers J, Maurer-Stroh S, Vanhee P, DopazoJz (2012). SNPeffect 4.0: on-line prediction of molecular and structural effects of protein-coding variants. Nucleic Acids Research.

[29] Kyte J, Doolittle RF (1982). A simple method for displaying the hydropathic character of a protein. Journal of Molecular Biology.

[30] Bergerat A, de Massy B, Gadelle D, Varoutas PC, Nicolas A, Forterre P (1997). An atypical topoisomerase II from Archaea with implications for meiotic recombination. Nature.

[31] Keeney S, Giroux CN, Kleckner N (1997). Meiosis-specific DNA double-strand breaks are catalyzed by SPO11, a member of a widely conserved protein family. Cell.

[32] Christensen GL, Ivanov IP, Atkins JF, Mielnik A, Schlegel PN, Carrell DT (2005). Screening the SPO11 and EIF5A2 genes in a population of infertile men. Fertility and Sterility.

[33] Ghalkhani E, Sheidai M, Gourabi H, Noormohammadi Z, Bakhtari N, Malekasgar AM (2014). Study of single nucleotide polymorphism (rs28368082) in SPO11 gene and its association with male infertility. Journal of Assisted Reproduction and Genetics.

[34] Nogués C, Fernández C, Rajmil O, Templado C (2009). Baseline expression profile of meiotic-specific genes in healthy fertile males. Fertility and Sterility.

[35] Székvölgyi L, Nicolas A (2010). From meiosis to postmeiotic events: homologous recombination is obligatory but flexible. The FEBS Journal.

[36] Shannon M, Richardson L, Christian A, Handel MA, Thelen MP (1999). Differential gene expression of mammalian SPO11/TOP6A homologs during meiosis. FEBS Letters.

[37] Romanienko PJ, Camerini-Otero RD (2000). The mouse SPO11 gene is required for meiotic chromosome synapsis. Molecular Cell.

[38] Liu Y, Wu C, Lyu Q, Yang D, Albertini DF, Keefe DL (2007). Germline stem cells and neo-oogenesis in the adult human ovary. Developmental Biology.

[39] Nouri N, Fazel-Najafabadi E, Behnam M, Nouri N, Aryani O, Ghasemi M (2014). Use of in silico tools for classification of novel missense mutations identified in dystrophin gene in developing countries. Gene.

[40] Alberts B, Johnson A, Lewis J, Raff M, Roberts K, Walter P (2002). Molecular Biology of the Cell.

[41] Venkatesh T, Suresh PS (2013). Exploration of deleterious single nucleotide polymorphisms in the components of human P bodies: an in silico approach. Gene.

[42] Shen LX, Basilion JP, Stanton VP Jr Single-nucleotide polymorphisms can cause different structural folds of mRNA. Proc Natl Acad Sci USA.

